# The Spiraling Case of a Yellow Chef: Isolated Hyperbilirubinemia

**DOI:** 10.1155/2018/5876718

**Published:** 2018-08-23

**Authors:** Karen Jiang, Ahmad Najdat Bazarbashi, Sami Dahdal, Adina Voiculescu, Natalia Khalaf

**Affiliations:** ^1^Division of Hospital Medicine, Department of Medicine, Brigham and Women's Hospital, Boston, MA, USA; ^2^Division of Gastroenterology, Hepatology and Endoscopy, Brigham and Women's Hospital, Boston, MA, USA; ^3^Department of Internal Medicine, St. John's Riverside Hospital, Yonkers, NY, USA; ^4^Division of Renal Medicine, Department of Medicine, Brigham and Women's Hospital, Boston, MA, USA

## Abstract

Leptospirosis is a common bacterial disease in tropical regions of the world due to greater exposure to rodents and domestic animals; however, this condition can also occur in US urban areas, though it often goes unrecognized. Gastrointestinal symptoms are very commonly seen, and icteric leptospirosis is often confused for other conditions resulting in delayed diagnosis and worse outcomes. As mortality increases with more extensive hepatic involvement, gastroenterologists should be aware of the constellation of gastrointestinal symptoms related to leptospirosis, as it can occur in the absence of classic exposure history.

## 1. Introduction

Leptospirosis is a prevalent zoonotic disease caused by the spirochete bacteria* Leptospira*. A number of mammals serve as natural hosts, with human infection occurring after exposure to animal or environmental contact. The disease is most common in tropical regions of the world, with an incidence in tropical climates being an estimated 10 times higher than in more temperate climates. However, cases of human infection in urban settings within developed countries such as the US have also been reported [1, 2]. Experts believe the disease to be underreported, with an estimated 873,000 cases of human infections occurring worldwide each year, accounting for an estimated 48,600 deaths [3].

Making a timely diagnosis of leptospirosis is challenging as its presentation can be diverse and nonspecific. The majority of affected individuals (75-100%) will suffer from fever, rigors, myalgias, and headache after an average incubation period of 10 days. Gastrointestinal symptoms such as nausea, vomiting, and diarrhea occur in up to 50% of cases, with less common symptoms including cough, sore throat, arthralgias, conjunctival suffusion, skin rash, abdominal pain, and aseptic meningitis [4–7].

It is important for gastroenterologists to recognize key features of leptospirosis, which commonly presents with gastrointestinal (GI) symptoms. Presented here is a case of icteric leptospirosis characterized by intrahepatic cholestasis and renal failure, highlighting the often key role gastroenterologists play in the care of these patients.

## 2. Case Presentation

A 56-year-old healthy man presented to the emergency department in the summer season with three days of fatigue and bilateral thigh pain. He was born in Puerto Rico but resided in the Northeast Region of the US, where he worked as a chef in a major metropolitan city. He had no sick contacts, recent travel, or alcohol or drug use.

Laboratory data on presentation demonstrated a creatinine (Cr) of 1.73 mg/dL, creatinine kinase (CK) of 3494 U/L and platelet count of 68x10^3^/*μ*L with initially normal liver function tests (LFTs). The patient was admitted for treatment of acute kidney injury from presumed rhabdomyolysis of unclear cause but subsequently developed low-grade fevers, leukocytosis, and worsening thrombocytopenia over the following days. His Cr worsened despite hydration and conservative management for which the patient underwent a renal biopsy on hospital day 4, with findings of acute tubular necrosis, interstitial hemorrhage, and capillaritis.

**Figure 1:**
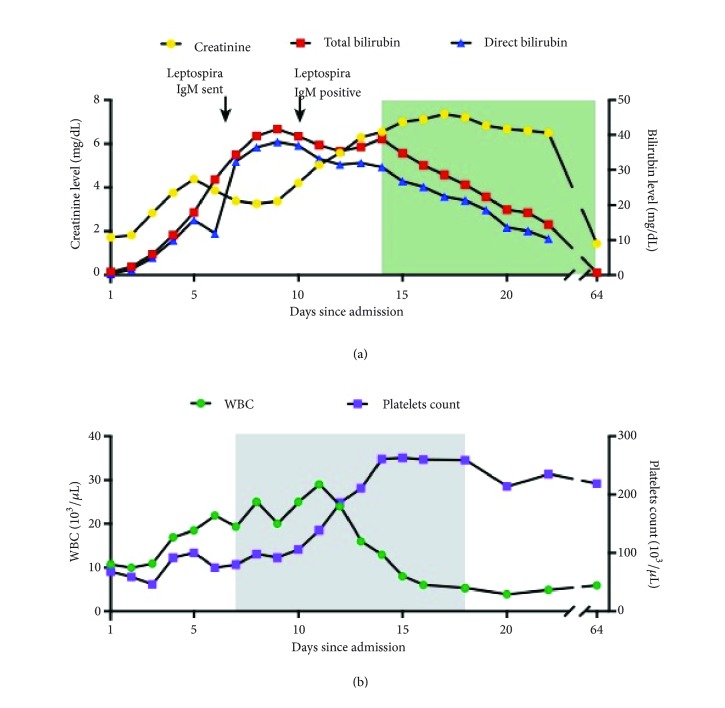
Multiorgan failure and response to treatments in patient with leptospirosis. (a) Creatinine, total bilirubin, and direct bilirubin levels during and after hospitalization. Shaded green area represents duration of cholestyramine and ursodiol treatment for bile cast nephropathy. (b) White-blood cell count (WBC) and platelet count during and after hospitalization. Shaded grey area represents duration of doxycycline treatment.

In addition to worsening renal function, he had an impressively rapid rise in his total and direct bilirubin with development of clinical jaundice over the subsequent days with laboratory values on hospital day 8 as follows: Cr of 4, total bilirubin of 41 mg/dL, and direct bilirubin of 38 mg/dL (Figure 1(a)). The GI consult service became involved in his care and on physical examination noted no evidence of chronic liver disease other than jaundice. The patient had no abdominal tenderness, hepatosplenomegaly, or asterixis. The bilirubin values were out of proportion to his other liver tests such as INR and albumin, which remained within normal values and AST/ALT and alkaline phosphatase values wavered between mildly elevated (<2 times the upper limit of normal) and normal values. Based on the kidney biopsy results and significant hyperbilirubinemia, testing was done for bacteremia, influenza, tuberculosis, HIV, tick-borne diseases, Hantavirus infection, acute viral hepatitis (A, B, C, E, CMV, EBV, and VZV) and vasculitis, which were all negative or normal.

An abdominal ultrasound and MRI liver protocol/MCRP showed a normal hepatobiliary system. Given his impressive rise in bilirubin out of proportion to other LFTs in combination with renal failure and rhabdomyolysis, the GI service recommended antibody testing for leptospirosis, for which serum IgM antibodies were checked on hospital day 5. The following day, he was started on empiric doxycycline in liaison with infectious disease consultation at 100 mg intravenously twice daily. In the interim, a liver biopsy was done showing liver parenchyma with marked canalicular and intracellular cholestasis, accentuated in perivenular zone, and rare foci of bile duct injury and ductular proliferation. There was no evidence of significant steatosis, fibrosis or intracellular iron deposition with trichrome, reticulin, PAS-D, and iron stains being unrevealing.

On hospital day 10, the Leptospira IgM returned positive, consistent with the diagnosis of icteric leptospirosis. He was continued on doxycycline 100 mg twice daily with subsequent normalization of leukocyte and platelet counts (Figure 1(b)). In view of the positive Leptospira IgM antibody results, the liver tissue obtained from biopsy was reevaluated for spirochete organisms with special staining; however no organisms were found.

The patient completed a 10-day course of doxycycline but unfortunately suffered from bile cast nephropathy from severe hyperbilirubinemia with continued rise in Cr (Figure 1(a)), for which he was treated with cholestyramine and ursodiol. His liver function tests and kidney laboratory results began to improve thereafter, and he was discharged on hospital day 26. At six-week follow-up, his renal function improved (Cr of 1.4 mg/dL) and his LFTs had normalized. It was later discovered that the restaurant where the patient had been working had a rodent infestation, which were the most likely source of his infection.

## 3. Discussion

Leptospirosis is the one of the most prevalent zoonotic diseases globally but is rare and often under recognized in developed countries. It is important for clinicians to be aware that sporadic cases of leptospirosis can be seen in urban areas where the disease is spread through the urine of rodents and domestic animals [8]. While some patients will only experience self-limited symptoms such as mild fever, myalgias, and fatigue, others suffer more severe disease states such as Weil's disease, as highlighted in the case. Weil's disease is characterized by multiorgan failure including liver failure, renal failure, and pulmonary hemorrhage. Other clinical manifestations include central nervous system and cardiac and ocular involvement [9]. Severe manifestations of leptospirosis are treated with antibiotics such as penicillin or doxycycline [10].

Recognizing the clinical manifestations of leptospirosis is critical for initiating treatment in a timely fashion. While the overall median mortality rate of leptospirosis is 2.2%, it is significantly more fatal once renal failure or jaundice develops, with mortality rates rising to 12% and 19%, respectively [10]. Gastroenterologists are often involved in the care of affected patients given the variety of GI symptoms people can develop, ranging from abdominal pain, vomiting, and diarrhea to more severe complications such as pancreatitis, acalculous cholecystitis, enteritis, peritonitis, and liver failure [11]. Diagnostic options for leptospirosis include serological testing such as agglutination testing and enzyme linked immunosorbent assays (ELISA); microscopic examination (dark field microscopy, silver staining, and Warthin-starry stain); molecular testing with PCR; and culture of organisms [12].

Our patient's presentation highlights the fact that leptospirosis can cause isolated direct hyperbilirubinemia or direct hyperbilirubinemia out of proportion to other LFT values. Jaundice in leptospirosis is a unique feature that remains incompletely understood; however intrahepatic cholestasis, duodenitis resulting in ampulla of Vater obstruction, and indirect hyperbilirubinemia from hemorrhage have all been described as causes of jaundice with this condition [13]. In mouse models, Leptospira bacteria have been seen to invade the intercellular junctions of host hepatocytes resulting in disruption of cellular junctions within bile canaliculi [14]. While awareness of the disease and a high clinical suspicion is required to identify this condition in a timely fashion, liver biopsy can be helpful, as this may reveal congested sinusoids, leptospiral attachment to and invasion of the perijunctional region between hepatocytes, and a lack of normal adhesion between hepatocytes with hepatocyte apoptosis. Damage to hepatocytes and disruption of their intercellular junction is thought to lead to bile leak from the canaliculi and into the sinusoidal blood vessels, thus causing direct hyperbilirubinemia [15]. The patient's liver biopsy revealed marked parenchymal and canalicular cholestasis, which can be seen with leptospirosis as well as a variety of other conditions. We hypothesize that the patient's liver biopsy did not reveal evidence of spirochetes on special staining because the patient was receiving treatment with empiric doxycycline prior to liver biopsy.

Treatment of leptospirosis depends on its clinical severity. Mild cases can be treated as an outpatient with penicillin, doxycycline, or azithromycin, with intravenous doxycycline, ceftriaxone, or cefotaxime recommended for more severe cases in hospitalized patients. Although not found to impact mortality, antimicrobial therapy may be associated with faster clinical resolution of symptoms [16]. Our patient was treated with doxycycline in discussion with our infectious disease colleagues given the broad differential diagnosis the patient had on admission including concern for tick-borne illnesses, for which doxycycline would be a preferred agent.

Our case is unique in that Weil's disease developed in a patient residing in an urban city in the US without obvious exposure history. Icteric leptospirosis can be confused for other hepatic conditions such as viral hepatitis, malaria, and sepsis [11] and should be considered in the differential diagnosis of patients presenting with both severe renal and liver injury, even in the absence of classic exposure history.

## References

[1] World Health Organization (2010). *Report of the First Meeting of the Leptospirosis Burden Epidemiology Reference Group, Geneva*.

[2] Hartskeerl R. A., Collares-Pereira M., Ellis W. A. (2011). Emergence, control and re-emerging leptospirosis: dynamics of infection in the changing world. *Clinical Microbiology and Infection*.

[3] World Health Organization Global burden of human leptospirosis and cross-sectoral interventions for its prevention and control. http://www.pmaconference.mahidol.ac.th/dmdocuments/2013-PMAC-Poster-P9-Bernadette%20Abela-Ridder.pdf.

[4] Vanasco N. B., Schmeling M. F., Lottersberger J., Costa F., Ko A. I., Tarabla H. D. (2008). Clinical characteristics and risk factors of human leptospirosis in Argentina (1999-2005). *Acta Tropica*.

[5] Sanford J. P. (1984). Leptospirosis—time for a booster. *The New England Journal of Medicine*.

[6] Berman S. J., Tsai C. C., Holmes K., Fresh J. W., Watten R. H. (1973). Sporadic anicteric leptospirosis in South Vietnam: a study in 150 patients. *Annals of Internal Medicine*.

[7] Katz A. R., Ansdell V. E., Effler P. V., Middleton C. R., Sasaki D. M. (2001). Assessment of the clinical presentation and treatment of 353 cases of laboratory-confirmed leptospirosis in hawaii 1974-1998. *Clinical Infectious Diseases*.

[8] Vinetz J. M., Glass G. E., Flexner C. E., Mueller P., Kaslow D. C. (1996). Sporadic urban leptospirosis. *Annals of Internal Medicine*.

[9] Vijayachari P., Sugunan A. P., Shriram A. N. (2008). Leptospirosis: an emerging global public health problem. *Journal of Biosciences*.

[10] Taylor A. J., Paris D. H., Newton P. N., Vinetz J. M. (2015). A systematic review of the mortality from untreated leptospirosis. *PLOS Neglected Tropical Diseases*.

[11] Vaishnavi C. (2013). *Infections of the Gastrointestinal System*.

[12] Levett P. N. (2001). Leptospirosis. *Clinical Microbiology Reviews*.

[13] Higgins R., Cousineau G. (1977). The pathogenisis of leptospirosis II. Jaundice in experimental leptospirosis in guinea pigs. *The Canadian Journal of Comparative Medicine*.

[14] Miyahara S., Saito M., Kanemaru T., Villanueva S. Y. A. M., Gloriani N. G., Yoshida S.-I. (2014). Destruction of the hepatocyte junction by intercellular invasion of Leptospira causes jaundice in a hamster model of Weil's disease. *International Journal of Clinical and Experimental Pathology*.

[15] Haake D. A., Levett P. N. (2015). Leptospirosis in humans. *Current Topics in Microbiology and Immunology*.

[16] Brett-Major D. M., Coldren R. (2012). Antibiotics for leptospirosis. *Cochrane Database of Systematic Reviews*.

